# Anti-Multiple Myeloma Potential of Secondary Metabolites from *Hibiscus sabdariffa*—Part 2

**DOI:** 10.3390/molecules26216596

**Published:** 2021-10-31

**Authors:** Alessio Malacrida, Valeria Cavalloro, Emanuela Martino, Giosuè Costa, Francesca Alessandra Ambrosio, Stefano Alcaro, Roberta Rigolio, Arianna Cassetti, Mariarosaria Miloso, Simona Collina

**Affiliations:** 1 School of Medicine and Surgery, University of Milan-Bicocca, 20900 Monza, Italy; alessio.malacrida@unimib.it (A.M.); roberta.rigolio@unimib.it (R.R.); 2 Experimental Neurology Unit, University of Milano-Bicocca, 20900 Monza, Italy; 3 Department of Earth and Environmental Sciences, University of Pavia, 27100 Pavia, Italy; valeria.cavalloro01@universitadipavia.it; 4 Department of Health Sciences, Campus “S. Venuta”, “Magna Græcia” University of Catanzaro, Viale Europa, 88100 Catanzaro, Italy; gcosta@unicz.it (G.C.); ambrosio@unicz.it (F.A.A.); alcaro@unicz.it (S.A.); 5 Net4Science Academic Spin-Off, Campus “S. Venuta”, “Magna Græcia” University of Catanzaro, Viale Europa, 88100 Catanzaro, Italy; 6 Associazione CRISEA-Centro di Ricerca e Servizi Avanzati per l’Innovazione Rurale, Località Condoleo di Belcastro (CZ), 88050 Belcastro, Italy; 7 CREA, Research Centre for Vegetable and Ornamental Crops, 18038 Sanremo, Italy; arianna.cassetti@crea.gov.it; 8 Department of Drug Sciences, University of Pavia, 27100 Pavia, Italy; simona.collina@unipv.it

**Keywords:** *Hibiscus sabdariffa*, *Hib-ester*, *Hib-carbaldehyde*, anthocyanins, proteasome, multiple myeloma, diet supplement, docking studies

## Abstract

Multiple Myeloma (MM) is an aggressive tumor causing millions of deaths every year and currently available therapies are often unsuccessful or correlated with severe side effects. In our previous work we demonstrated that the *Hibiscus sabdariffa* hydroalcoholic extract inhibits the growth of the MM cell line and we isolated two metabolites responsible for the activity: *Hib-ester* and *Hib-carbaldehyde*. Herein we report their interaction with proteasome, one of the main targets in the fight against MM. The molecular modelling study outlined a good interaction of both compounds with the target and these results prompted us to investigate their potential to inhibit proteasome. Metabolites were then isolated from the calyces and an extract with a high content of *Hib-ester* and *Hib-carbaldehyde* was prepared. An anticancer profile was drawn, evaluating apoptosis, autophagy and proteasome inhibition, with the anticancer properties being mainly attributed to the *Hib-ester* and *Hib-carbaldehyde*, while the proteasome inhibition of the extract could also be ascribed to the presence of anthocyanins, a class of secondary metabolites already known for their proteasome inhibitory activity.

## 1. Introduction

Multiple myeloma (MM) is a neoplastic disease causing renal dysfunction, hypercalcemia and lytic lesions in the bone [1,2]. About 80% of patients at the time of diagnosis are also affected by myeloma bone disease (MBD), caused by an imbalance of the bone remodeling process in the bone marrow microenvironment. This imbalance induces the formation of osteolytic lesions that reduce bone structural integrity and often culminate in pathological fractures. MM is due to an increased protein turnover as well as to an overproduction of monoclonal and misfolded proteins, whereas a complex and not fully elucidated cell–cell interaction between MM tumor cells is responsible for MBD [3,4,5].

Despite different therapeutic options that are available, MM is considered an unmet neoplastic disease since it causes millions of deaths every year. Moreover, drugs currently available often lead to drug resistance and severe side effects, therefore, specific therapeutic options for MBD that are able to stimulate osteoblastic activity could represent an important supportive strategy to treat MM.

To date, the gold standard therapies of MM are drugs acting as proteasome inhibitors. Proteasome is a multicatalytic complex responsible for 80–90% of protein degradation. It is also involved in the degradation of IkB, the inhibitor of nuclear factor kappa B (NFkB), allowing NFkB to induce the inflammatory process, to promote e neoangiogenesis as well as the proliferation, migration, and suppression of apoptosis during the tumor process [6].

**Figure 1:**
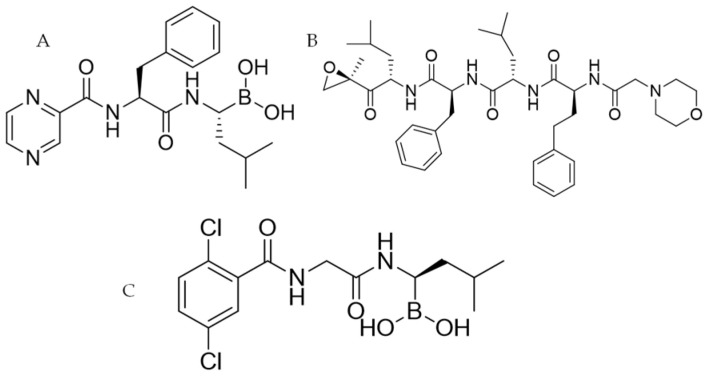
2D structure of the FDA-approved proteasome inhibitors BTZ (**A**), CFZ (**B**) and Ixazomib (**C**).

Currently, the following proteasome inhibitors are available for clinical use [6,7]:

- Bortezomib (Velcade^®^, BTZ: Figure 1A): the first proteasome inhibitor FDA-approved acting against Multiple myeloma (MM) and Mantle cell lymphoma [8]. BTZ is a dipeptide boronic acid and its reversible effect is due to the bond between its boronic group and the proteasome’s β5-subunit [9].
- Carfilzomib (Kyprolis^®^, CFZ: Figure 1B): this second-generation inhibitor belongs to the epoxyketone class and it has the same binding site as BTZ, but it delivers an irreversible effect. It has been approved to treat MM, but it is also studied for use against leukemia, amyloidosis, lymphoma and macroglobulinemia [10].
- Ixazomib (Ninlaro^®^: Figure 1C): belongs to the same class of drug as BTZ and has the same mechanism of action. Ixazomib also presents significant clinical advantages including an increased oral bioavailability. The anti-MM therapy with Ixazomib needs a combination with Dexamethasone and Lenalidomide [11].

The above-mentioned proteasome inhibitors may be unsuccessful or linked to severe side effects including, among others, neurotoxicity. More than a half of the patients treated with BTZ suffer from this side effect, thus requiring a dose reduction or complete suspension of the therapy [12]. Therefore, efforts are needed for finding new proteasome inhibitors characterized by higher efficiency, better tolerability, and lower toxicity [13,14].

**Figure 2:**
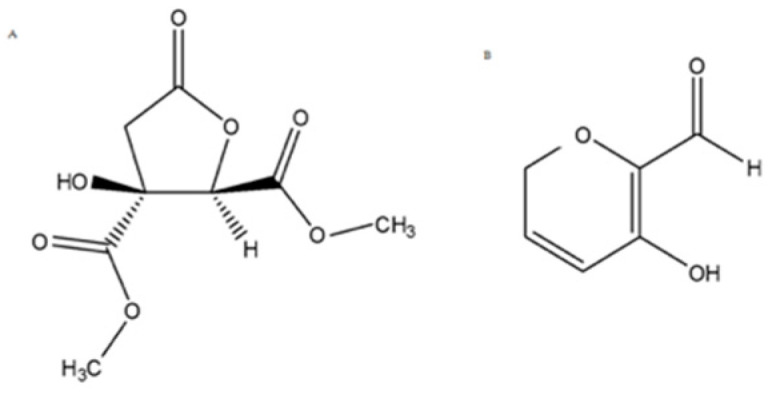
2D structure of the isolated secondary metabolites *Hib-ester* (**A**) and *Hib-carbaldehyde* (**B**).

Our interdisciplinary research group has been active in this field for several years [15,16]. Recently we studied *Hibiscus sabdariffa* L., an herbaceous subshrub also known as karkade. Calyces of karkade are commonly used by the food industry as antioxidants, food colorants, and as a good source of phytochemicals [17]. We demonstrated the potential of *H. sabdariffa* flower extract as an anti-MM agent, and we isolated and identified two of its secondary metabolites effective against the MM cell line at non-neurotoxic concentrations (Figure 2) [18]. 

In the present work, molecular modeling studies were carried out to investigate the binding mode and the theoretical binding affinity of *Hib-ester* and *Hib-carbaldehyde* (Figure 2) versus the proteasome. Then, the anticancer properties and mechanism of action of the hydroalcoholic *H. sabdariffa* extract and of the main metabolites were deepened.

## 2. Results and discussion

### 2.1. Molecular Modelling Studies

To investigate the ability of the *Hib-ester* and *Hib-carbaldehyde* compounds to recognize and bind the proteasome active site, molecular modeling studies were carried out.

Molecular analysis highlighted that the two *H. sabdariffa* metabolites were well accommodated in the proteasome chymotrypsin-like site, presenting a good theoretical binding affinity (Table 1).

**Figure 3:**
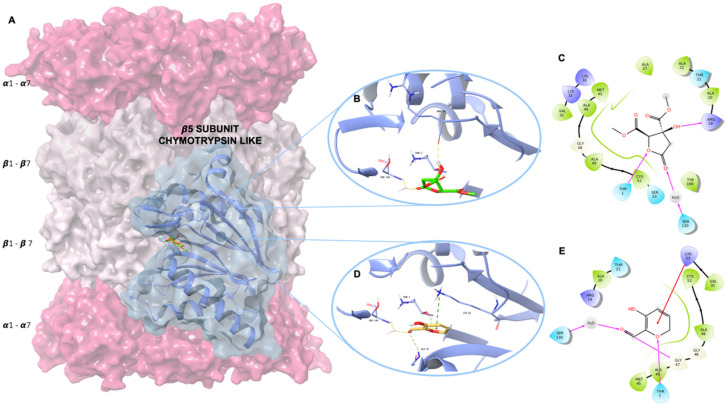
3D representation of (**A**) human 20S proteasome with a focus on the β5 chymotrypsin like subunit; (**B**) *Hib-ester* and (**D**) *Hib-carbaldehyde* docked into the proteasome chymotrypsin-like binding pocket. The *Hib-ester* and the *Hib-carbaldehyde* are depicted, respectively, as green and yellow carbon sticks, the proteasome is shown as a light-blue cartoon and the enzyme residues involved in crucial contacts with the compounds are reported as light-blue carbon sticks. The water molecule is shown as red carbon sticks. Hydrogen bonds and pi-cation contacts are shown, respectively, as dashed yellow and dark-green lines. 2D representation of (**C**) *Hib-ester* and (**E**) *Hib-carbaldehyde* complexed into the proteasome chymotrypsin-like binding pocket. In the 2D representation, hydrogen bonds and pi-cation contacts are shown, respectively, as violet and red lines.

By analyzing the binding modes of the lowest energy pose of the two compounds, we observed that both compounds were involved in productive interactions with the proteasome chymotrypsin active site. By using the Maestro graphical interface contact analysis [19] we observed that the two metabolites strongly interact with the binding pocket residues by means of a hydrogen bond, hydrophobic and pi-cation interactions (Figure 3). The *Hib-ester* establishes hydrogen bond interactions with the Arg19 and Thr1, and a water bridge with the Ser130 proteasome residues (Figure 3B,C). Moreover, the *Hib-ester* is engaged in several hydrophobic contacts with Thr1, Arg19, Ala20, Thr21, Lys33, Met45, Ala46, Gly47 and Ala49. Regarding *Hib-carbaldehyde*, we found that the ligand is able to establish a pi-cation interaction with the Lys33, hydrogen bond interactions with the Thr1 and Gly47, and a water bridge with the Ser130 of the proteasome (Figure 3D,E). In addition, the *Hib-carbaldehyde* was found to be well stabilized in the chymotrypsin site by means of several hydrophobic contacts with Thr1, Arg19, Ala20, Thr21, Val 31, Lys33, Met45, Ala46, Gly47, Gly48 and Ala 49. Our modeling results elucidate that both *Hib-ester* and *Hib-carbaldehyde* are able to interact with the main chains of the same pivotal residues of the proteasome chymotrypsin pocket.

To assess the actual ability of *Hib-ester* and *Hib-carbaldehyde* to bind the proteasome active site, as predicted by the in silico studies, experimental analyses were carried out.

### 2.2. Extraction of H. sabdariffa

To obtain the *Hib-ester* and the *Hib-carbaldehyde* in suitable amounts for an extensive biological investigation, we optimized the extraction procedure previously applied [18].

The dried and powdered *H. sabdariffa* calyces were subjected to a microwave assisted solvent extraction (MASE) procedure, using ethanol 80% as the extracting solvent and heating to 60 °C. This method was selected both because MASE allows for a high efficiency to be obtained (low extraction time with high extraction yields), and because ethanol is considered a green solvent [20,21,22]. Moreover, the applied conditions were selected taking into account the stability of the major constituents of the extracts (anthocyanins), being the resulting raw extract endowed with antioxidant activity, as reported in our previous contribution [20]. The obtained ethanolic extract was then fractionated, optimizing our preliminary results. In detail, we performed a liquid/liquid extraction, suspending the extract in water and extracting with Ethyl Acetate. The dried organic layers were evaporated and considered as an enriched fraction of the extract (HsEF). The HsEF was analyzed with respect to its total anthocyanin content (TAC). The TAC was quantified according to the well consolidated pH-differential method [23,24,25] where the measurements were conducted depending on the pH change (pH 1.0 and pH 4.5). The results evidenced that 3 mg/mL of HsEF contains 7.2 μg of cyanidin-3-O-glucoside, representing 0.23% of the total extract.

Subsequently, the enriched fraction was further fractionated as follows:

- HsEF was treated with polymer-supported carbonate (PS-carbonate) resin in methanol. After solvent removal, the resin was subjected to treatment with 0.1% HCl in methanol, filtrated and the solvent evaporated under a reduced pressure to obtain a simplified fraction. It was further purified by flash chromatography, thus obtaining *Hib-ester* (final yield = 0.22%) (Figure 2A).
- HsEF was subjected to a second liquid/liquid extraction using dichloromethane as the organic solvent and renewing the aqueous phase during the process. This second phase was then evaporated and chromatographed to obtain pure *Hib-carbaldehyde*, with a 1.1% yield (Figure 2B).

### 2.3. Cell Viability and Cell Death of Myeloma Cell Lines

The cytotoxicity of the HsEF was evaluated in comparison with the isolated metabolites in RPMI 8226 and U266.B1 myeloma cell lines. The cells were treated with increasing concentrations of HsEF, *Hib-ester* and *Hib-carbaldehyde* for 24, 48 and 72 h.

**Figure 4:**
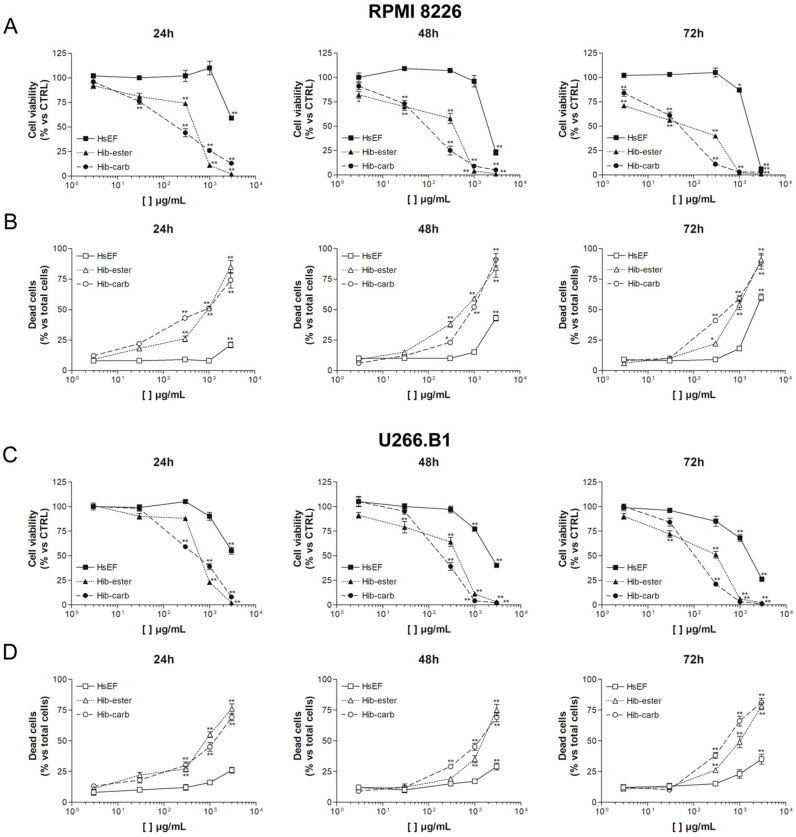
Trypan blue cell viability and death of RPMI 8226 and U266.B1 cells. (**A**) Cell viability and (**B**) cell death of RPMI 8226 cells treated with increasing concentrations of HsEF, *Hib-ester* and *Hib-carbaldehyde* (3 µg/mL–3 mg/mL), and counted with trypan blue after 24, 48 and 72 h. (**C**) Cell viability and (**D**) cell death of U266.B1 cells treated with increasing concentrations of HsEF, *Hib-ester* and *Hib-carbaldehyde* (3 µg/mL–3 mg/mL), and counted with trypan blue after 24, 48 and 72 h. Cell viability data are represented as the mean percentage ± SD and are compared to untreated controls, arbitrarily set to 100%. Cell death data are represented as the mean percentage ± SD calculated on the sum of all counted cells for each treatment (* *p* < 0.05, ** *p* < 0.01 vs. CTRL).

Results of a trypan blue vital count assay highlighted that the HsEF impaired the cell viability of both cell lines in a time dependent manner, with a particular effect on the RPMI 8226 cells (IC_50_ at 24 h≃3 mg/mL). This effect was mainly due to the *Hib-ester* and *Hib-carbaldehyde*, having an IC_50_ of 450 µg/mL and 200 µg/mL, respectively. Moreover, after 72 h of treatment with the HsEF 3 mg/mL, 61% of the RPMI 8226 cells and 35% of the U266.B1 cells were dead (Figure 4).

Being that the RPMI 8226 cells were more sensitive, this cell line was chosen for subsequent studies. The concentrations of the compounds were instead chosen on the basis of the IC_50_ obtained at 24 h of treatment (HsEF 3 mg/mL, *Hib-ester* 450 µg/mL (2.1 mM) and *Hib-carbaldehyde* 200 µg/mL (1.6 mM)) (Table 2).

### 2.4. Evaluation of Apoptosis

To understand if this cytotoxicity was driven by necrosis or apoptosis, an Annexin V assay and cleaved caspase 3 Western blotting were performed [26].

**Figure 5:**
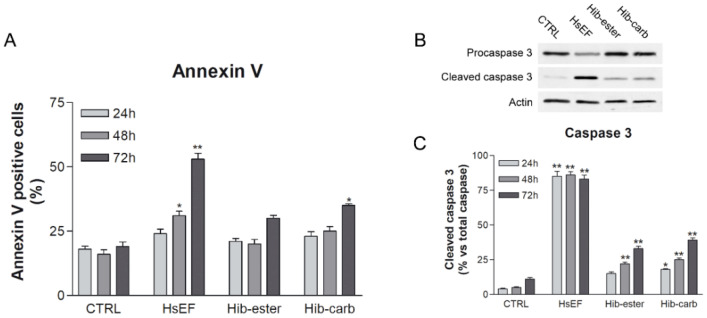
RPMI 8226 cells apoptosis. (**A**) Annexin V positive cells after treatment with HsEF 3 mg/mL, *Hib-ester* 450 µg/mL and *Hib-carbaldehyde* 200 µg/mL for 24, 48 and 72 h. (**B**) Representative images and (**C**) quantification of caspase 3 Western blotting. Data are represented as the mean percentage ± SD, compared to untreated RPMI 8226 cells (CTRL) (* *p* < 0.05, ** *p* < 0.01 vs. CTRL).

Untreated RPMI 8226 cells presented an Annexin positivity of 18%, consistent with the mortality observed on the trypan blue count, and a cleaved caspase 3 of about 4–10%. Annexin V positive RPMI 8226 cells significantly increased in a time dependent manner only after HsEF treatment. The *Hib-carbaldehyde* treatment presented a significant percentage of positive cells only after 72 h of treatment. For the *Hib-ester* treatment, no significant differences were observed compared to the control cells at all examined times (Figure 5). Moreover, no significant increase was observed in PI-only positive cells treated with HsEF (Figure S1).

Furthermore, the HsEF induced a significant cleavage of caspase 3 at all evaluated time points and the percentage of cleaved caspase 3 was more than 80%, compared to total caspase 3. The *Hib-ester* and *Hib-carbaldehyde* induced a significant increase in cleaved caspase 3 compared to untreated RPMI 8226 cells. For both compounds the increase of cleaved caspase 3 was much lower than for the HsEF (Figure 5).

Both these experiments evidenced that the considered enriched fraction is able to induce apoptosis in the treated cells. Even if the *Hib-ester* and *Hib-carbaldehyde* induced a similar increase in cleaved caspase 3 and in Annexin V positive cells, their effect is lower than the HsEF.

### 2.5. Evaluation of Autophagy Inhibition

We then evaluated the autophagy, one of the main cellular degradation systems responsible for the induction of cell death, survival, but also drug resistance in cancer [27,28]. It is often upregulated after proteasome impairment by drugs such as BTZ [29]. To evaluate if the HsEF, *Hib-ester*, and *Hib-carbaldehyde* could affect autophagy, autophagy Acridine Orange staining and Western blot analysis of autophagy markers was performed. In detail, we evaluated the levels of Beclin1, which has a central role in the regulation of autophagy [30], and LC3, a soluble protein recruited to autophagosomal membranes during the autophagy process [29].

**Figure 6:**
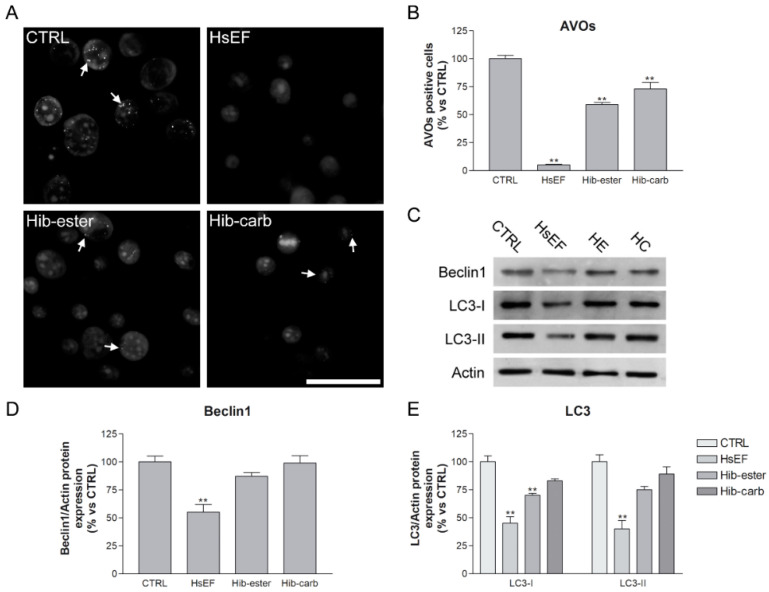
Autophagy inhibition (**A**) representative images of RPMI 8226 cells stained with Acridine Orange. Cells were not treated (CTRL) or treated with HsEF 3 mg/mL, *Hib-ester* 450 µg/mL and *Hib-carbaldehyde* 200 µg/mL for 24 h. (**B**) Red fluorescence of Acridine Orange images was quantified and data were represented in the graph as the percentage of AVOs positive cells compared to untreated controls (arbitrarily set to 100%). (**C**) Representative images of Western blots of Beclin1, LC3-I, LC3-II and actin. RPMI cells were not treated (CTRL) or treated with HsEF 3 mg/mL, *Hib-ester* 450 µg/mL and *Hib-carbaldehyde* 200 µg/mL for 24 h. (**D**,**E**) Quantification of Western blots of Beclin1, LC3-I and LC3-II. Data are expressed as mean percentage ± SD compared to untreated controls, arbitrarily set to 100%. (** *p* < 0.01 vs. CTRL).

Compared to the untreated cells, which had a detectable physiological autophagic activity, the HsEF treatment significantly reduced the percentage of acidic vesicular organelles (AVOs, lysosomes and autophagolysosomes stained with Acridine Orange). In addition, the *Hib-ester* and *Hib-carbaldehyde* reduced the AVOs, but the reduction was much lower than for the HsEF (Figure 6A,B).

Moreover, after treatments with HsEF, the Beclin1 expression level was significantly reduced compared to untreated RPMI 8226 cells. Instead, treatment with the *Hib-ester* and *Hib-carbaldehyde* induced an insignificant, slight reduction compared to the untreated RPMI 8226 cells.

After RPMI 8226 treatment with the HsEF, the expression of both the cytosolic form (LC3-I) and the autophagosomal membrane form (LC3-II), was significantly reduced compared to untreated cells. *Hib-carbaldehyde* did not significantly reduce either the LC3-I or LC3-II protein expression. *Hib-ester* significantly reduced the LC3-I expression, but to a lesser extent than the HsEF, while the LC3-II protein level was only slightly decreased by the *Hib-ester*.

### 2.6. Evaluation of Proteasome Inhibition

The capability of the HsEF, *Hib-ester* and *Hib-carbaldehyde* to inhibit proteasome has been evaluated. The RPMI 8226 cells were treated with HsEF 3 mg/mL, *Hib-ester* 450 µg/mL and *Hib-carbaldehyde* 200 µg/mL, and after 24 h the proteasome activity was evaluated. Both the extract and its metabolites were able to significantly impair proteasome activity compared to untreated RPMI 8226 cells.

**Figure 7:**
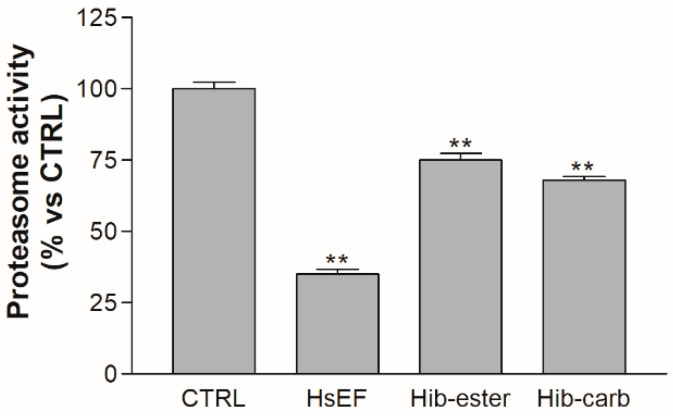
Proteasome activity of RPMI 8226 cells. The graph represents proteasome activity of RPMI 8226 cells, not treated (CTRL) or treated with HsEF 3 mg/mL, *Hib-ester* 450 µg/mL and *Hib-carbaldehyde* 200 µg/mL for 24 h. Data are represented as the mean percentage ± SD, compared to CTRL, arbitrarily set to 100%. (** *p* < 0.01 vs. CTRL).

Interestingly, the HsEF resulted in being much more effective than both the *Hib-ester* and *Hib-carbaldehyde*, inducing a 65% reduction in proteasome activity at 3 mg/mL, compared to the *Hib-ester* and *Hib-carbaldehyde* effect of 25% and 30% reduction, respectively (Figure 7). This higher activity could be due to the anthocyanins content of the *H. sabdariffa* ethanolic extract, which represented 0.23%. These are secondary metabolites with an already known proteasome inhibitory activity [31,32]. Consistently, the proteasome inhibition activity demonstrated by the HsEF was the result of the activity of anthocyanins, *Hib-ester* and *Hib-carbaldehyde* together.

## 3. Materials and Methods

### 3.1. General

HPLC grade solvents were supplied by Honeywell (Germany), while analytical grade solvents were supplied by PanReac (Germany). The evaporation procedures were performed under reduced pressure using a Heidolph Laborota 4000 instrument (Heidolph Instruments GmbH & Co., Schwabach, Germany).

Analytical thin-layer chromatography (TLC) was carried out on silica gel pre-coated glass-backed plates (Fluka Kieselgel 60 F254, Merck, Darmstadt, Germany). The detection was conducted with UV light or ceric ammonium molybdate (IV) stain (Hanessian’s Stain).

Flash chromatography was performed with silica gel 60 (particle size 230–400 mesh) purchased from Nova Chimica (Cinisello Balsamo, Italy).

Fetal bovine serum (FBS; Gibco) was purchased from ThermoFisher Scientific (Lisbon, Portugal).

*H. sabdariffa* calyces were powdered using a blade mill (A10 IKA-Werke GmbH & Co., Staufen, Germany) and then extracted exploiting a multimode microwave apparatus (MARSX press, CEM Corporation, Matthews, NC, USA).

### 3.2. Molecular Modeling Analysis

Starting from the crystal structure of the human 20S proteasome complex with Ixazomib, deposited in the Protein Data Bank (PDB) with the PDB code 5LF7 [33], our molecular modeling analysis was carried out.

The receptor structure was prepared by means of the Protein Preparation Wizard tool implemented in Maestro, using OLPS_2005 as the force field. Residual crystallographic buffer components were removed, missing side chains were built using the Prime module, hydrogen atoms were added, and side chain protonation states at pH 7.4 were assigned [34].

In order to evaluate the reliability of our molecular recognition approach, we performed redocking calculations by using the Glide Standard Protocol (SP) algorithm [35] that was able to reproduce the experimentally determined binding mode. In fact, we obtained a root mean square deviation (RMSD) value equal to 0.651 Å.

The compounds were prepared by means of a LigPrep tool [36], hydrogens were added, salts were removed, ionization states were calculated using an ionizer at pH 7.4 and then all the compounds were submitted to a MacroModel energy minimization, using OPLS_2005 as the force field [37].

The docking studies were performed by means of a Glide v. 6.7 SP algorithm [35] and 10 poses for the ligands were generated.

### 3.3. Pan Assay Interference Compounds (PAINS) Evaluation

The PAINS properties of the chemical structures of the investigated compounds were theoretically investigated by means of the ZINC PAINS Pattern Identifier web server [38]. The method used did not highlight any PAINS related to the *Hib-ester* and *Hib-carbaldehyde* compounds.

### 3.4. Extraction Procedure

#### 3.4.1. Plant Material

*H. sabdariffa* calyces were stored in dark conditions and, at the time of use, cut to a small size and grounded with a blade mill. Then, 10 g of the so obtained homogeneous fine powder was dispersed in 200 mL of Ethanol 80% and subjected to microwave heating (2 min ramping, 5 min hold time, maximum pressure 120 psi, maximum potency 400 W, temperature 60 °C, 3 cycles). The mixture was left to cool at room temperature, filtrated, concentrated under reduced pressure to remove the alcoholic solvent and then extracted five times with ethyl acetate. The combined organic phases were dried with anhydrous Na_2_SO_4_ and the solvent removed under a reduced pressure to obtain the *H. sabdariffa* enriched fraction (HsEF) (2.5 g, 25% yield).

The HsEF was spectrophotometrically analyzed with respect to total anthocyanin content (TAC) at 510–700 nm using a UV–Visible spectrophotometer (UV–Visible Spectracomp 620, Advanced Products, Milan, Italy) [23].

A 200 µL solution of 3 mg/mL of HsEF was mixed with 800 µL of a KCl buffer (0.025 M, pH 1.0). The absorbance of the mixture was measured at 510 and 700 nm using distilled water to zero the spectrophotometer. Another aliquot of the same volume of HsEF was then combined with a CH_3_COONa buffer (0.4 M, pH 4.5), and the absorbance was measured at the same wavelengths. The absorbance of the diluted sample (A) was calculated using Equation (1):

A = (A_λ510_ − A_700_)_pH1.0_ − (A_λ510_ − A_700_) _pH4.5_ (1)

Next, the TAC was calculated using Equation (2):

(2) $$\mathrm{mg}\;\mathrm{cyanidin}-3-\mathrm{glucoside}/L=\frac{A\;\times \;\mathrm{MW}\;\times \;\mathrm{DF}\;\times \;1000}{\varepsilon \;\times \;l}$$

A = absorbance of the diluted sample; DF = dilution factor; MW = 484.83; ε = 26,900; l = length of the cell; The quantitative result was given in mg cyanidin-3-glucoside [30].

#### 3.4.2. Isolation of *Hib-ester*

The HsEF (2.5 g) was resolubilized in methanol (50 mL) and then subjected to a treatment with PS-carbonate (5 g). The mixture was shaken at room temperature for 1 h, then filtered and washed with methanol (30 mL) and dichloromethane (150 mL). The resin was then recovered and 50 mL of MeOH + 0.1% HCl was added. The system was shaken for 3 h at room temperature, and the solvent filtered. The procedure was performed twice. The combined methanolic filtrates were evaporated under reduced pressure and then subjected to a flash chromatography on silica gel with 50% Toluene, 30% hexane and 20% isopropyl alcohol as the mobile phase. Finally, the *Hib-ester* (TLC: RF = 0.41) was isolated and its structure confirmed comparing its nuclear magnetic resonance (1H- and 13C-NMR) and mass spectra with those obtained in our previous work [18].

ESI-MS: *m*/*z* 218 [M + H]^+^. ^1^H-NMR (CDCl_3_, 400 MHz): δH: 5.1 (1H, s), 3.9 (3H, s), 3.8 (3H, s), 3.0 (1H, d, *J* = 17.4 Hz), 2.8 (1H, d, *J* = 17.4 Hz). ^13^C-NMR (CDCl_3_): δC: 172, 168, 165, 82, 77, 55, 54, 40.

#### 3.4.3. Isolation of *Hib-carbaldehyde*

The HsEF (2.5 g) was dissolved in 100 mL of water and then washed three times with dichloromethane. The aqueous phase was concentrated in vacuo and then subjected to a flash chromatography on silica gel with 70% dichloromethane and 30% ethyl acetate as the mobile phase. The collected fractions corresponded to pure *Hib-carbaldehyde* (TLC: RF = 0.35, yellow oil). Its structure was confirmed comparing its nuclear magnetic resonance (1H- and 13C-NMR) and mass spectra with those obtained in our previous work [18].

ESI-MS: *m*/*z* 127 [M + H]^+^, 149 [M + Na]^+^. ^1^H-NMR (CDCl_3_, 400 MHz): δH: 9.6 (1H, s), 7.2 (1H, m), 6.4 (1H, m), 4.6 (2H, d). ^13^C-NMR (CDCl_3_): δC: 178, 160, 155, 125, 110, 58.

### 3.5. Biological Assays

#### 3.5.1. Cell Cultures and *Hibiscus sabdariffa*

Multiple myeloma RPMI 8226 and U266.B1 cells were cultured in an RPMI 1640 medium supplemented with 10% fetal bovine serum, 1% L-Glutamine and 1% Penicillin and Streptomycin (EuroClone, Pero, Italy).

The HsEF, *Hib-ester* and *Hib-carbaldehyde* were solubilized in PBS at 1 g/mL concentration. Further working dilutions were made directly in the culture medium [39].

#### 3.5.2. Trypan Blue Vital Count Assay

Cells were seeded in 6-well plates at 2.5 × 103 cells/well density and were treated after 24 h with increasing concentrations of HsEF, *Hib-ester* or *Hib-carbaldehyde*. Untreated cells represented the controls. After 24, 48 and 72 h, the cells were collected, stained with Trypan Blue dye (Sigma Aldrich, St. Louis, MO, USA) and counted in a hemocytometer. Both viable and dead cells were counted. The percentage of death cells was calculated on the sum of all counted cells.

#### 3.5.3. Annexin V Assay

The RPMI 8226 cells were seeded and treated as described in paragraph 4.2. After 24, 48 and 72 h, the cells were collected and washed two times with PBS. 1 × 105 cells were incubated for 15 min at 37 °C in 100 µL of a 1X Annexin binding buffer and 10 µL of an Annexin V and propidium iodide solution (Sigma Aldrich, St. Louis, MO, USA). At the end of incubation, 400 µL of the 1X Annexin V binding buffer was added to the solution and the cells were analyzed by a flow cytometer (FACS Canto, BD Biosciences, Franklin Lakes, NJ, USA).

#### 3.5.4. Western Blotting

Protein total extracts were obtained from RPMI 8226 cells treated with HsEF, *Hib-ester* or *Hib-carbaldehyde* for 24, 48 and 72 h. Protein total extracts were prepared also from untreated cells, that represented the controls. The cells were harvested, washed three times with PBS and resuspended in 100 µL of a Lysis Buffer (Hepes 5 mM pH 7.5, NaCl 150 mM, Glycerol 10%, Triton X100 1%, MgCl_2_ 1.5 mM, EGTA 5 mM, PMSF 4 nM, Aprotinin 1%, Sodium pyrophosphate 20 nM and Sodium orthovanadate 92 mg/mL). Cell lysates were vortexed for 30 s and clarified with a centrifuge at 13,500 RPM for 15 min at 4 °C. Protein contents were quantified using a Bradford assay.

An amount of 10 µg of the protein samples were added to a Laemmli buffer and then denatured at 95 °C for 5 min. The proteins were separated in a 13% acrylamide SDS-PAGE. After electrophoresis, the proteins were transferred to nitrocellulose filters and Western blot (WB) analysis was performed.

Membranes’ blocking, washing and antibody incubation were performed according to manufacturer’s instructions. Antibodies against LC3B (1:1000, Cell Signaling, Danvers, MA, USA), Beclin-1 (1:1000 Cell Signaling, Danvers, MA, USA), pro-Caspase-3 (1:1000, Santa Cruz Biotechnology, Dallas, TX, USA), cleaved Caspase 3 (1:1000, Cell Signaling, Danvers, MA, USA) and beta actin (1:1000, Santa Cruz Biotechnology, Dallas, TX, USA), were used. After incubation with primary antibodies, the membrane was washed and then incubated with appropriate horseradish peroxidase-conjugated secondary antibodies (1:2000) (anti-mouse, Merck Life Science, Milano, Italy; anti-rabbit, PerkinElmer, Milano, Italy). Immunoreactive proteins were visualized using an ECL chemiluminescence system (Euroclone, Pero, Italy).

#### 3.5.5. Acridine Orange Staining

RPMI 8226 cells were seeded at a density of 2.5 × 105 cells/well in 6-well plates and after 24 h they were treated with HsEF, *Hib-ester* or *Hib-carbaldehyde*. After 24 h the cells were stained with Acridine Orange (AO) (Merck Life Science, Milano, Italy) at a final concentration of 0.5 µg/mL for 15 min at 37 °C. The cells were then washed 3 times with PBS and divided for FACs analysis or fluorescent microscopy imaging. For the FACs analysis the cells were suspended in PBS and analyzed (excitation 488 nm, emission 510 nm for green; excitation 460 nm, emission 650 nm for red). For microscopy imaging the cells were placed on a slide and observed under a fluorescent microscope.

#### 3.5.6. Proteasome Activity Assay

RPMI 8226 cells were seeded, treated and lysed as described in paragraph 3.5.4, but the lysis buffer was prepared without proteases and phosphatases inhibitors.

An amount of 40 µg of proteins, 10 µL of 10× proteasome buffer (Hepes pH 7.5 250 mM, EDTA pH 8.0 5 mM, NP-40 0.5%, SDS 0.01%) and 10 µL of proteasome substrate (N-Succinyl-Leu-Leu-Val-Tyr-7-Amido-4-Methylcoumarin, 7.6 mg/mL) (Merck Life Science, Milano, Italy), were loaded in each well of a black 96-well plate. After 2 h at 37 °C, the fluorescence was quantified in a microplate reader (excitation 380 nm, emission 460 nm) (BMG-Labtech, Ortenberg, Germany).

#### 3.5.7. Statistical Analysis

Data were reported as mean ± standard deviation (SD) from at least three independent experiments. Statistical analysis was performed using GraphPad Prism 3 software. The differences between the control and treated cells were evaluated using a one-way ANOVA analysis of variance followed by Dunnett’s multiple comparison test. Statistical significance was set at *p* < 0.05 or *p* < 0.01.

## 4. Conclusions

In the present work, we moved a step forward in evaluating the potential of *H. sabdariffa* against MM. Since one of the most important mechanisms of action of anti-MM drugs is the proteasome inhibition, we firstly investigated in silico the capability of *Hib-ester* and *Hib-carbaldehyde* to interact with proteasome. Our modeling results evidenced that both the compounds are able to interact with the residues of the proteasome chymotrypsin pocket. Moreover, neither the *Hib-ester* nor the *Hib-carbaldehyde* resulted in PAINS, as highlighted by the ZINC PAINS Pattern Identifier, therefore, encouraged by these results, we deepened the biological investigation. A microwave assisted solvent extraction (MASE) procedure was applied, the ethanolic extract was then simplified via a liquid/liquid extraction, thus obtaining the HsEF fraction, characterized by the presence of 0.23% of anthocyanins, and determined by applying the well consolidated pH-differential method [23,24,25]. The HsEF was further purified to dispose of *Hib-ester* and *Hib-carbaldehyde* in suitable amounts for further investigation.

The cytotoxic effect of the HsEF, *Hib-ester and Hib-carbaldehyde* on the human MM cell line (RPMI 8226) was then evaluated. The HsEF, *Hib-ester and Hib-carbaldehyde* were endowed with cytotoxic activity, with the *Hib-carbaldehyde* being the most effective one. To confirm the mechanism of the actions of the HsEF, *Hib-ester* and *Hib-carbaldehyde,* apoptosis, proteasome inhibition and autophagy activity were evaluated. The results obtained confirmed the proteasome inhibition activity of the *Hib-ester and Hib-carbaldehyde.* Nevertheless, the HsEF was revealed to be more effective in inducing apoptosis, inhibiting proteasome and reducing autophagy. These results suggest that a synergic effect occurs in the presence of other metabolites existing in the extract and we hypothesized that anthocyanins played a key role in the HsEF activity.

Further experiments focused on the isolation and identification of metabolites responsible for the HsEF activity as well as the elucidation of the biological mechanisms of the *Hib-ester and Hib-carbaldehyde* are ongoing.

## Figures and Tables

**Table 1:** Docking score values calculated for the two *Hibiscus sabdariffa* metabolites in complex with the proteasome.

Compounds	Docking Score *
*Hib-ester*	−5.62
*Hib-carbaldehyde*	−6.18

* Docking score values are expressed in kcal/mol.

**Table 2:** IC_50_ of RPMI 8226 and U266.B1 cells after treatment with HsEF, HE and HC.

**RPMI 8226**			
**µg/mL**	**IC_50_ 24 h**	**IC_50_ 48 h**	**IC_50_ 72 h**
HsEF	>3000	2356 ± 418	1634 ± 115
*Hib-ester*	454 ± 57	319 ± 38	35 ± 2
*Hib-carbaldehyde*	208 ± 10	85 ± 8	38 ± 8
**U266.B1**			
**µg/mL**	**IC_50_ 24 h**	**IC_50_ 48 h**	**IC_50_ 72 h**
HsEF	>3000	2497 ± 88	1837 ± 134
*Hib-ester*	640 ± 37	387 ± 62	272 ± 55
*Hib-carbaldehyde*	460 ± 75	207 ± 27	115 ± 14

## Data Availability

Not applicable.

## Supplementary Materials

The following are available online, Figure S1: Annexin V and Propidium Iodide staining of RPMI 8226 cells. (A) Representative dot-plots of FACS analysis of RPMI 8226 cells treated with HsEF or not treated for 24, 48 and 72 h, and stained with Annexin V and Propidium Iodide. (B) Graphs represent the percentage of cells negative to Annexin V and Propidium Iodide (Neg), positive only to Annexin V (AV), positive only to Propidium Iodide (PI), or positive to both Annexin V and Propidium Iodide (AV+PI). *p* < 0.01 vs. CTRL.
