# Supplementary material for: Anti-Multiple Myeloma Potential of Secondary Metabolites from Hibiscus sabdariffa—Part 2
Source: Molecules. 2021 Oct 31;26(21):6596. doi: 10.3390/molecules26216596 (PMC8588054; doi:10.3390/molecules26216596)
Supplement: Supplementary file 1 [file molecules-26-06596-s001.zip › molecules-1434427-supplementary.pdf]

Anti-Multiple Myeloma Potential of Secondary metabolites from *Hibiscus sabdariffa* - Part 2

Alessio Malacrida, Valeria Cavalloro, Emanuela Martino, Giosuè Costa, Francesca Alessandra Ambrosio, Stefano Alcaro, Roberta Rigolio, Arianna Cassetti, Mariarosaria Miloso, and Simona Collina

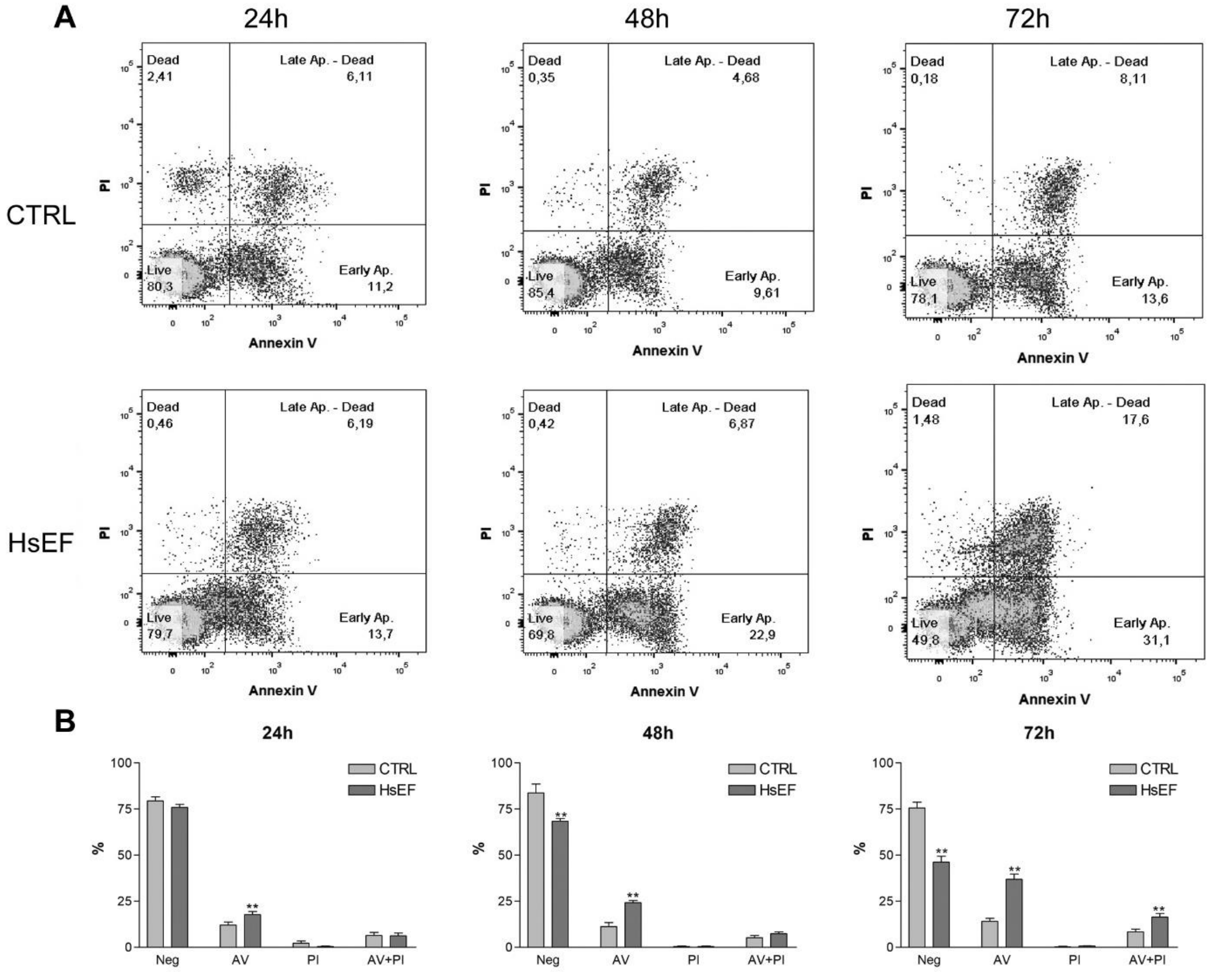

Supplementary Figure S1. Annexin V and Propidium Iodide staining of RPMI 8226 cells. (A) Representative dot-plots of FACS analysis of RPMI 8226 cells treated with HsEF or not treated for 24, 48 and 72 hours, and stained with Annexin V and Propidium Iodide. (B) Graphs represent the percentage of cells negative to Annexin V and Propidium Iodide (Neg), positive only to Annexin V (AV), positive only to Propidium Iodide (PI), or positive to both Annexin V and Propidium Iodide (AV+PI). $p < 0.01$ vs CTRL.
